# The effect of selection and referral biases for the treatment of localised prostate cancer with surgery or radiation

**DOI:** 10.1038/s41416-018-0071-4

**Published:** 2018-03-29

**Authors:** Christopher J. D. Wallis, Gerard Morton, Sender Herschorn, Ronald T. Kodama, Girish S. Kulkarni, Sree Appu, Bobby Shayegan, Roger Buckley, Arthur Grabowski, Steven A. Narod, Robert K. Nam

**Affiliations:** 1 Division of Urology, Sunnybrook Health Sciences Centre, University of Toronto, Toronto, Canada; 2 Department of Radiation Oncology, Sunnybrook Health Sciences Centre, University of Toronto, Toronto, Canada; 3 Division of Urology, University Health Network, University of Toronto, Toronto, Canada; 4 Division of Urology, St. Josephs Healthcare, McMaster University, Hamilton, Canada; 5 Division of Urology, North York General Hospital, University of Toronto, Toronto, Canada; 6 Division of Urology, Durham Regional Cancer Centre, University of Toronto, Toronto, Canada; 7 Department of Public Health Sciences, University of Toronto, Toronto, Canada

**Keywords:** Surgical oncology, Health policy

## Abstract

**Background:**

Consultation with radiation oncologists, in addition to urologists, is advocated for patients diagnosed with prostate cancer. Treatment patterns for patients receiving consultation from radiation oncologists in addition to urologists have not previously been described.

**Methods:**

We conducted a matched cohort study of men with newly diagnosed non-metastatic prostate cancer in Ontario, Canada. Patients who underwent consultation with a radiation oncologist prior to treatment were matched 1:1 with patients managed by a urologist alone based on tumour and patient characteristics. We examined rates of active treatment (surgery or radiotherapy) within one year following diagnosis.

**Results:**

Among 5708 matched pairs (11,416 patients), those who received radiation oncology consultation were more likely to undergo active treatments whether they had intermediate or high-risk disease (88.6% vs. 65.9%, *p* < 0.0001; adjusted odds ratio 4.0, 95% CI: 3.6–4.4) or low-risk disease (56.1% vs. 13.3%, *p* < 0.0001; adjusted odds ratio 8.4, 95% CI: 6.7–10.6). This effect persisted after considering age, comorbidity, tumour volume and year of diagnosis.

**Conclusions:**

Patients newly diagnosed with prostate cancer who receive radiation oncology consultation are associated with a higher rate of active treatment, compared to patients managed by urologists only. Selection and referral biases, and unmeasured confounding such as patient preference must be considered as important factors attributing this association.

## Introduction

In most cases, the diagnosis of clinically localised prostate cancer is first established by a urologist who communicates treatment options with the patient, including radical prostatectomy, radiotherapy, and active surveillance.^1^ Consultation with a radiation oncologist is typically made based, in part, on the characteristics of the tumour as well as the patient’s age, comorbidity, and urologist and patient preference.^2^

Guidelines of several organisations including the National Comprehensive Cancer Network have recommended that all patients with newly diagnosed clinically localised prostate cancer be assessed by both a urologist and a radiation oncologist prior to treatment,^3, 4^ and this has been proposed as a quality metric.^5^ In Ontario, Cancer Care Ontario (CCO), a branch of the Provincial government which funds all cancer treatment, recommended that all patients should receive consultation from both a urologist and radiation oncologist prior to treatment.^4^

The effect of selection and referral biases on treatment patterns of prostate cancer patients receiving consultation from both urologists and radiation oncologists compared to urologists alone is inherent in treatment decision-making. Given that new guidelines are mandating consultation with both a urologist and radiation oncologists, we sought to quantify and characterise the impact on treatment patterns for patients undergoing a radiation oncology consultation.

## Materials and methods

We conducted a population-based, retrospective cohort study of men diagnosed with non-metastatic prostate cancer in Ontario, Canada between 2010 and 2013. In Ontario, medical care is reimbursed through a single, government-funded health insurance system (Ontario Health Insurance Plan, OHIP). Patients were identified in the Ontario Cancer Registry (OCR). We selected 2010 for cohort inception as reliable linkage to biopsy pathology and staging data were available beginning at this time.

The Sunnybrook Health Sciences Centre REB approved this study which was conducted and reported according to the STROBE guidelines^6^ and the RECORD statement.^7^

### Outcome

The primary outcome was receipt of active treatment with surgery or radiotherapy in the year following diagnosis. We used OHIP physician billings and procedural codes from the Canadian Institute of Health Information (CIHI) Discharge Abstract Database and National Ambulatory Care Reporting System^8^ to identify radical prostatectomy or radiotherapy (Supplementary Table 1). Any patient who did not receive surgery or radiotherapy in the year following diagnosis was categorised as receiving conservative management. Patients in the Urologist alone group could receive radiotherapy if their radiation oncology consultation occurred after the 90-day time period (*n* = 667 in primary analysis).

### Baseline characteristics and covariates

Linked health administrative data were used to capture a wide variety of baseline characteristics including tumour factors, demographics, general and specific comorbidities, and general medical care. Data on tumour grade (Gleason score) and stage were derived from the OCR and serum prostate-specific antigen (PSA) levels were determined from the Ontario Laboratory Information System. General comorbidity was measured using the Resource utilisation Band (RUB), a well-established measure of comorbidity,^9^ based on the Johns Hopkins Aggregate Disease Groups.^10^

Due to administrative delays in data capture, tumour stage and grade were missing for 13,204 patients and PSA data were missing for 3608 patients. These patients were excluded. Patients retained and those excluded were similar in demographic characteristics and comorbidities (significant differences <0.1) though excluded patients were more likely to be diagnosed in 2012/2013.

Consultation with a radiation oncologist (RadOnc) was identified as the date of consultation occurring after the date of prostate cancer diagnosis. We identified physician interactions following diagnosis using the OHIP database.^11^ We identified radiation oncologist visits within 90 days of prostate cancer diagnosis in order to capture an initial “therapeutic decision” window, and performed sensitivity analyses varying the length of this period. Control patients were managed by a Urologist alone.

### Statistical analysis

We matched patients who received radiation oncology consultation with those who did not using a combination of variables and the propensity score. Patients were directly matched on age at diagnosis, socioeconomic status, index year of diagnosis, prostate cancer risk category, and the propensity score. Prostate cancer risk was categorised as low risk (Gleason ≤6, Stage 1, and PSA <10 ng/mL) and intermediate/high risk (Gleason score ≥7, Stage 2 disease, or serum PSA ≥10 ng/mL). Stage 1 and 2 are patients with no evidence of metastases. The propensity score was derived from a multivariable logistic regression model for receiving radiation oncologist consultation using the remaining baseline characteristics as predictors, including geographic region, number of general practitioner (GP) visits, number of hospitalisations, ever resident of long-term care, RUB score, diabetes, myocardial infarction, cerebrovascular accident, congestive heart failure, chronic obstructive pulmonary disease, hypertension, arrhythmia, dementia, liver disease and renal disease. Patients in the RadOnc group were matched to those in the Urologist alone group 1:1 using the greedy algorithm. standardised differences were used to compare baseline characteristics of the two groups with differences of <0.1 (10%) considered acceptable balance.^12^

Due to the clustering inherent in matched data, we used generalised estimating equations with a logit wing to determine the effect of radiation oncologist consultation on receipt of active treatment. The matching identifier was used as a clustering variable. Statistical analyses were performed using SAS 9.3 (SAS Institute Inc., Cary, NC, USA).

### Subgroup and exploratory analyses

We performed a number of pre-planned exploratory analyses based on clinically relevant subgroups in which the effect of radiation oncologist consultation on receipt of active treatment may differ, including prostate cancer risk, age and comorbidity, date of diagnosis, location of residence (urban or rural), and tumour volume. We performed an additional subgroup analysis assessing the effect of same-day consultation with a urologist and a radiation oncologist.

In addition to assessing the role of radiation oncologist consultation, we examined the effect of visits with primary care physicians and medical oncologists on the receipt of active treatment.

We performed a tracer analysis to assess for the influence of health or intervention seeking behaviours using cataract surgery and cholecystectomy as tracer outcomes.^13^

## Results

We identified 16,666 men newly diagnosed with non-metastatic prostate cancer who had complete pathology data and met inclusion criteria. Of those, 9587 (57.5%) were managed by a urologist alone and 7079 (42.5%) received a radiation oncologist consultation within 90 days of diagnosis. Of these patients, 640 (9.0%) were seen concurrently by a urologist and radiation oncologist (i.e., on the same day). Patients who received a radiation oncologist consultation were older, and were more likely to have intermediate or high-risk prostate cancer (Gleason score ≥7, Stage 2 disease, or serum PSA ≥10 ng/mL) than patients managed by a urologist alone (Table 1). Other baseline characteristics were similar between the two groups (Table 1).

**Table 1:** Baseline characteristics before and after propensity score matching by multidisciplinary assessment group

	Before matching			After matching		
	RadOnc (*n* = 7079)	Urologist alone (*n* = 9587)	standardised difference	RadOnc (*n* = 5708)	Urologist alone (*n* = 5708)	standardised difference
Demographics						
Age (median, IQR)	67 (62–73)	65 (59–71)	0.22	67 (61–72)	66 (61–72)	0.01
*Index year (n, %)*			0.07			0.00
2010	2150 (30.4)	3038 (31.7)		1806 (31.6)	1806 (31.6)	
2011	2506 (35.4)	3484 (36.3)		2065 (36.2)	2065 (36.2)	
2012	1699 (24.0)	2321 (24.2)		1377 (24.1)	1377 (24.1)	
2013	724 (10.2)	744 (7.8)		460 (8.1)	460 (8.1)	
*Income quintile (n, %)*			0.03			0.00
1 (lowest)	985 (14.3)	1449 (15.1)		852 (14.9)	852 (14.9)	
2	1234 (17.7)	1755 (18.3)		1034 (18.1)	1034 (18.1)	
3	1383 (19.5)	1876 (19.6)		1086 (19.0)	1086 (19.0)	
4	1619 (22.5)	2124 (22.2)		1271 (22.3)	1271 (22.3)	
5 (highest)	1832 (25.6)	2353 (24.5)		1465 (25.7)	1465 (25.7)	
Missing	26 (0.4)	30 (0.3)		0	0	
*Geographic region (n, %)*			0.08			0.01
Region 1	333 (4.7)	425 (4.4)		281 (4.9)	287 (5.0)	
Region 2	605 (8.5)	822 (8.6)		518 (9.1)	528 (9.3)	
Region 3	254 (3.6)	542 (5.7)		217 (3.8)	224 (3.9)	
Region 4	824 (11.6)	1348 (14.1)		725 (12.7)	696 (12.2)	
Region 5	298 (4.2)	521 (5.4)		259 (4.5)	263 (4.6)	
Region 6	481 (6.8)	825 (8.6)		421 (7.4)	431 (7.6)	
Region 7	465 (6.6)	679 (7.1)		410 (7.2)	406 (7.1)	
Region 8	911 (12.9)	1103 (11.5)		755 (13.2)	733 (12.8)	
Region 9	783 (11.1)	1213 (12.7)		689 (12.1)	726 (12.7)	
Region 10	322 (4.5)	390 (4.1)		274 (4.8)	260 (4.6)	
Region 11	1045 (14.8)	713 (7.4)		560 (9.8)	528 (9.3)	
Region 12	257 (3.6)	424 (4.4)		224 (3.9)	231 (4.0)	
Region 13	339 (4.8)	476 (5.0)		299 (5.2)	315 (5.5)	
Region 14	154 (2.2)	100 (1.0)		76 (1.3)	80 (1.4)	
Tumour-related factors						
*Prostate cancer risk (n, %)*			0.31			0.00
Low risk	1029 (14.5)	2591 (27.0)		909 (15.9)	909 (15.9)	
Not low risk	6050 (85.5)	6996 (73.0)		4799 (84.1)	4799 (84.1)	
*Serum PSA level*						
Median (ng/mL) (IQR)	7 (5-11)	6 (5–9)	0.27	7 (5-11)	7 (5-10)	0.16
Categorical			0.21			0.11
<10 ng/mL	4957 (70.0)	7583 (79.1)		4066 (71.2)	4334 (75.9)	
≥10 ng/mL	2122 (30.0)	2004 (20.9)		1642 (28.8)	1374 (24.1)	
*Gleason score (n, %)*			0.35			0.18
≤6	1892 (26.7)	4717 (49.2)		1619 (28.4)	2331 (40.8)	
7	3810 (53.8)	3934 (41.0)		3046 (53.4)	2714 (47.5)	
≥8	1377 (19.5)	936 (9.8)		1043 (18.3)	663 (11.6)	
*Stage (n, %)*			0.29			0.10
1	1069 (15.1)	2731 (28.5)		943 (16.5)	1002 (17.6)	
2	5092 (71.9)	5410 (56.4)		4028 (70.6)	3734 (65.4)	
3	918 (13.0)	1446 (15.1)		737 (12.9)	972 (17.0)	
General medical care						
Number of GP visits in year prior to diagnosis (median, IQR)	5 (3–9)	5 (3–9)	0.06	5 (3–9)	5 (3–9)	0.00
hospitalisation in year prior to diagnosis (*n*, %)	463 (6.5)	617 (6.4)	0.00	347 (6.1)	346 (6.1)	0.00
Ever resident of long-term care prior to diagnosis (*n*, %)	6 (0.1)	12 (0.3)	0.01	<5	<5	0.01
Comorbidity						
*RUB score (n, %)*			0.02			0.01
0	<5	<5		0	<5	
1	<5	2–6		<5	<5	
2	250 (3.5)	403 (4.2)		215–219	205 (3.6)	
3	4455 (62.9)	6162 (64.3)		3678 (64.4)	3673 (64.3)	
4	1574 (22.2)	1997 (20.8)		1207 (21.1)	1232 (21.6)	
5	796 (11.2)	1018 (10.6)		603 (10.6)	594 (10.4)	
Diabetes diagnosis (*n*, %)	1680 (23.7)	2055 (21.4)	0.05	1287 (22.5)	1303 (22.8)	0.01
MI in 5 years prior (*n*, %)	94 (1.3)	104 (1.1)	0.02	67 (1.2)	62 (1.1)	0.01
CVA in 5 years prior (*n*, %)	38 (0.5)	25 (0.3)	0.04	17 (0.3)	12 (0.2)	0.02
History of CHF (*n*, %)	302 (4.3)	365 (3.8)	0.02	230 (4.0)	214 (3.7)	0.01
History of COPD (*n*, %)	1072 (15.1)	1359 (14.2)	0.03	836 (14.6)	827 (14.5)	0.00
History of hypertension (*n*, %)	4287 (60.6)	5481 (57.2)	0.07	3372 (59.1)	3432 (60.1)	0.02
History of arrhythmia (*n*, %)	52 (0.7)	57 (0.6)	0.02	38 (0.7)	38 (0.7)	0.00
History of dementia (*n*, %)	105 (1.5)	171 (1.8)	0.02	70 (1.2)	76 (1.3)	0.01
History of liver disease (*n*, %)	23 (0.3)	38 (0.4)	0.01	20 (0.4)	21 (0.4)	0.00
History of renal disease (*n*, %)	175 (2.5)	248 (2.6)	0.01	137 (2.4)	130 (2.3)	0.01

Note: in order to comply with ICES privacy regulations, cell sizes smaller than 5 were suppressed. Where cells <5 would be calculable, the next smallest cell in the category was also suppressed. Note: Socioeconomic status was assessed using a standardised technique based on the income quintile of the patient’s neighborhood of residence. Geographic region was characterised using local health integration networks (LHINs), regions that plan and deliver health-care resources and thus affect a patient’s access to care^20^

*GP* general practitioner, *RUB* resource utilisation band, a measure of comorbidity derived from the Johns Hopkins ADG system, *MI* myocardial infarction, *CVA* cerebrovascular accident, *CHF* congestive heart failure, *COPD* chronic obstructive pulmonary disease

Among the patients who received a radiation oncologist consultation, 84.1% (*n* = 5951) received active treatment (radical prostatectomy or radiation), compared to 52.1% (*n* = 4996) of those treated by a urologist alone (*p* < 0.0001; Table 2). The unadjusted odds ratio for receiving active treatment associated with radiation oncologist consultation was 5.72 (95% CI 5.43–6.02). Of the patients who saw a radiation oncologist (RadOnc group), 1733 (24.5%) had surgery, 4218 (59.6%) had radiation, and 1128 (15.9%) had conservative management, whereas of the patients who were treated by the urologist along (Uro group), 4329 (45.2%) had surgery, 667 (6.9%) had radiation, and 4591 (47.9%) had conservative management. Patients in the Uro group may have received radiotherapy within a year of diagnosis if their consultation with a radiation oncologist occurred following the 90-day “therapeutic decision” window.

**Table 2:** Treatment allocation for patients in study cohort

	Cohort prior to matching (*n* = 16,666)		Cohort after matching (*n* = 11,416)	
	RadOnc (*n*, %)	Urologist alone (*n*, %)	RadOnc (*n*, %)	Urologist alone (*n*, %)
Whole cohort				
No treatment	1128 (15.9)	4591 (47.9)	948 (16.6)	2423 (42.5)
Radiation	4218 (59.6)	667 (6.9)	3311 (58.0)	496 (8.7)
EBRT	3474 (49.1)	523 (5.5)	2647 (46.4)	414 (7.3)
Brachytherapy^a^	744 (10.5)	144 (1.5)	664 (11.6)	82 (1.4)
Surgery	1733 (24.5)	4329 (45.2)	1449 (25.4)	2789 (48.9)
Patients with low-risk disease (Gleason ≤6, Stage 1, and PSA <10 ng/mL)				
No treatment	448 (43.5)	2293 (88.5)	399 (43.9)	789 (86.8)
Radiation	512 (49.8)	113 (4.4)	450 (49.5)	48 (0.8)
EBRT	212 (20.6)	43 (1.7)	183 (20.1)	15 (1.7)
Brachytherapy^a^	300 (29.2)	70 (2.7)	267 (29.4)	33 (3.6)
Surgery	69 (6.7)	185 (7.1)	60 (6.6)	72 (7.9)
Patients with intermediate/high-risk disease (Gleason score ≥7, Stage 2 disease, or serum PSA ≥10 ng/mL)				
No treatment	680 (11.2)	2298 (32.9)	549 (11.4)	1634 (34.1)
Radiation	3706 (61.3)	554 (7.9)	2861 (59.6)	448 (7.8)
EBRT	3262 (53.9)	480 (6.9)	2464 (51.3)	399 (8.3)
Brachytherapy^a^	444 (7.3)	74 (1.1)	397 (8.3)	49 (1.0)
Surgery	1664 (27.5)	4144 (59.2)	1389 (28.9)	2717 (56.6)

*EBRT* external beam radiotherapy

^a^ Includes both low-dose rate (LDR) and high-dose rate (HDR) brachytherapy

The patients in the matched analysis are compared in Table 1 and the matching yielded adequate balances between the two groups. In the analysis of 5708 matched pairs, 83.4% of patients in the RadOnc group underwent active treatment within a year of diagnosis, compared to 57.5% of patients in the Uro group (*p* < 0.0001; Table 2; OR = 3.70, 95% CI 3.42–4.01; Table 3).

**Table 3:** Effect of radiation oncologist consultation (compared with urologist management alone) on the odds of active treatment (as compared to no treatment) within the year following prostate cancer diagnosis, among a number of clinical relevant subgroups

	Whole cohort		Low-risk prostate cancer (Gleason ≤6, Stage 1, and PSA <10 ng/mL)		Intermediate or high-risk prostate cancer	
	Odds ratio	95% CI	Odds ratio	95% CI	Odds ratio	95% CI
Primary analysis						
Unmatched cohort	5.72	5.43–6.02	9.98	8.40–11.86	3.86	3.52–4.24
Matched cohort	3.70	3.42–4.01	8.40	6.65–10.62	4.00	3.60–4.44
Subgroup analysis of age and comorbidity						
Age <70; comorbidity ≤3	2.87	2.57–3.20	7.21	5.81–8.96	2.16	1.85–2.52
Age <70; comorbidity 4 or 5	3.86	3.23–4.62	10.44	7.6–15.23	2.42	1.91–3.07
Age >70; comorbidity ≤3	16.59	13.73–20.05	30.83	16.65–57.08	13.49	10.95–16.61
Age >70; comorbidity 4 or 5	20.18	16.35–24.89	23.77	11.72–48.21	17.53	13.95–22.02
Subgroup analysis of region of residence						
Urban	4.72	4.36–5.12	9.70	8.05–11.69	3.76	3.40–4.15
Rural	6.10	4.87–7.65	12.65	7.83–20.14	4.98	3.75–6.61
Subgroup analysis of patients undergoing initial biopsy at an academic facility						
	4.52	3.91–5.24	8.99	6.25–12.94		
Subgroup analysis of tumour volume among those with low-risk prostate cancer						
Low volume			4.46	2.14–9.31		
High volume			4.93	2.07–11.74		
Subgroup analysis of patients receiving MDA on the same day						
Urologist alone	referent		Referent		Referent	
Same-day RadOnc	3.61	2.96–4.39	6.01	4.07–8.87	3.41	2.62–4.43
Asynchronous RadOnc	5.01	4.63–5.42	10.64	8.90–12.72	3.91	3.55–4.31

We stratified patients by prostate cancer risk, based on grade, stage, and serum PSA. For patients who had low-risk prostate cancer (Gleason score ≤6, Stage 1, and serum PSA <10 ng/mL), the odds ratio for undergoing active treatment for patients in the RadOnc group was significantly higher (adjusted OR 8.40, 95% CI 6.65–10.62), compared to those in the Uro group. For patients in the intermediate/high-risk group (Gleason score ≥7, Stage ≥2, or serum PSA ≥10 ng/mL), the adjusted odds ratio for active treatment was 4.00 (95% CI: 3.60–4.44). The frequency of specific treatments is outlined in Table 2.

In order to better understand the association between radiation oncologist consultation and the probability of active treatment, we conducted additional exploratory analyses. We stratified patients into four groups based on age (<70 or ≥70) and comorbidity (RUB score ≤3 or ≥4). Among patients with low-risk disease, we found that the effect of radiation oncologist consultation on treatment was largest for older patients and those with greater comorbidity (odds ratio for treatment up to 30.8 (95% CI: 16.7–57.1), Table 3). Over time, the use of conservative management increased for patients with low-risk disease in the Uro group, but not in the RadOnc group, over time (Table 4). In patients with intermediate/high-risk disease, the use of conservative treatment decreased over time among patients in the RadOnc group but not in the Uro group. We further examined a subgroup of patients with low-risk prostate cancer for whom information on tumour volume information was available (*n* = 291; 8.0% of low-risk cohort). These patients were stratified into those with low-volume disease (≤2 positive cores; *n* = 195) or high-volume disease (≥3 positive cores; *n* = 96).^3^ The RadOnc group was associated with a significantly increased likelihood of active treatment both in patients with low-volume, low-risk disease, and high-volume, low-risk disease (Table 3). In both subgroups, radiation oncologist consultation was associated with higher use of radiotherapy and lower use of conservative management (Table 5).

**Table 4:** Proportion of patients receiving no active treatment in the year following diagnosis, over time

	Overall cohort		RadOnc		Urologist alone	
	No treatment (*n*, %)	Cohort	No treatment (*n*, %)	Cohort	No treatment (*n*, %)	Cohort
Low-risk prostate cancer (Gleason ≤6, Stage 1 and PSA <10 ng/mL)						
2010	813 (70.5)	1154	153 (41.0)	373	660 (84.5)	781
2011	992 (75.4)	1316	163 (43.7)	373	829 (87.9)	943
2012	690 (82.5)	836	93 (48.4)	192	597 (92.7)	644
2013	246 (78.3)	314	39 (42.9)	91	207 (92.8)	223
Trend^a^	Increase (*p* < 0.001)		No trend (*p* = 0.25)		Increase (*p* < 0.001)	
Intermediate or high-risk prostate cancer (Gleason ≥7 or Stage ≥2, or PSA ≥10 ng/mL)						
2010	988 (24.5)	4034	219 (12.3)	1777	769 (34.1)	2257
2011	1070 (22.9)	4674	251 (11.8)	2133	819 (32.2)	2541
2012	711 (22.3)	3184	162 (10.8)	1507	549 (32.7)	1677
2013	209 (18.1)	1154	48 (7.1)	633	161 (30.9)	521
Trend^a^	Decrease (*p* < 0.001)		Decrease (*p* = 0.002)		No change (*p* = 0.17)	

^a^ Trend assessed using Cochran-Armitage test for trend

**Table 5:** Treatment allocation for patients in the subgroup of patients with low-risk prostate cancer (Gleason ≤6, Stage 1, and PSA <10 ng/mL) and available data on biopsy tumour volume (*n* = 291), stratified by volume of prostate cancer

	RadOnc (*n*, %)	Urologist alone (*n*, %)
Low-volume prostate cancer (≤2 positive cores)		
No treatment	38 (62.3)	118 (88.1)
Any treatment	23 (37.7)	16 (11.9)
Radiation	16 (26.2)	<5
EBRT	10 (16.4)	0
Brachytherapy^a^	6 (9.8)	*<5*
Surgery	7 (11.5)	11-15
High-volume prostate cancer (≥3 positive cores)		
No treatment	17 (34.0)	33 (71.7)
Any treatment	33 (66.0)	13 (28.3)
Radiation	26 (52.0)	7 (15.3)
EBRT	13 (26.0)	<5
Brachytherapy^a^	13 (26.0)	<5
Surgery	7 (14.0)	6 (13.0)

Note: in order to comply with ICES privacy regulations, cell sizes smaller than 5 were suppressed. Where cells <5 would be calculable, the next smallest cell in the category was also suppressed

*EBRT* external beam radiotherapy

^a^ Includes both low-dose rate (LDR) and high-dose rate (HDR) brachytherapy

Also, we found no significant changes in our results when stratified by academic centres (where biopsies are reviewed by specialised genitourinary pathologists), or when there were same-day radiation and urology consultations (Table 3, Supplementary Tables 2 and 3). Notably, compared to patients who received asynchronous radiation oncology consultation, those who had consultation on the same day had much higher utilisation of conservative management and surgery for intermediate and high-risk disease (Supplementary Table 3).

In the 90 days following diagnosis, 12,769 (76.6%) of patients in the matched cohort had at least one visit with a primary care physician (PCP) and 123 (0.7%) received consultation with a medical oncologist. In a multivariable model assessing the effect of radiation oncologist consultation, medical oncology consultation and visits with PCPs, radiation oncologist consultation remained the most prominent predictor of active treatment (OR 3.70, 95% CI 3.42–4.01, *p* < 0.0001). Visiting with a PCP was associated with an increased likelihood of receiving active treatment (OR 1.20, 95% CI 1.09–1.32, *p* = 0.0002) while consultation with a medical oncologist had no effect (OR 0.84, 95% 0.53–1.35, *p* = 0.48).

From tracer analysis to assess for health or intervention-seeking behaviours, we did not find a difference in rates of cataract removal (*p* = 0.18) or cholecystectomy (*p* = 0.76) between patients receiving radiation oncologist consultation and those who did not. The results of our primary analysis did not substantively differ when the window for radiation oncology consultation from diagnosis varied from 60 (OR 4.15, 95% CI 3.81–4.47) to 120 (OR 5.11, 95% CI 4.75–5.50) or 180 days (OR 5.38, 95% CI 5.00–5.78).

## Discussion

In Ontario, approximately one half of patients with non-metastatic prostate cancer receive consultation with a radiation oncologist prior to initiating treatment. After matching, we found that consultation with a radiation oncologist was associated with increased active treatment (83% vs. 58%) and decreased use of conservative management (17% vs. 43%). This discrepancy was present for both intermediate/high- and low-risk disease, but higher among patients with low-risk disease, with the majority undergoing radiotherapy. These findings were similar and consistent in several subgroups defined by various host and tumour factors, suggesting a strong presence of selection and referral biases.

To our knowledge, this is the first study that characterises the impact of treatment patterns among patients undergoing a multidisciplinary assessment prior to prostate cancer treatment. In this study, we have exhaustively evaluated for potential confounding and effect modification in treatment rates, including patient age at diagnosis, comorbidity, year of diagnosis, prostate cancer risk category, tumour volume among those men with low-risk disease, and those diagnosed at academic facilities. While the magnitude of effect varied within these subgroups, radiation oncologist consultation was consistently associated with an increased odds of active treatment. Also, referral to a radiation oncologist increased the proportion of patients undergoing radiation treatment for both low and intermediate/high-risk disease categories. This could reflect the intent of multidisciplinary assessments by providing patients with informed choices among those who desire treatment leading to more patient-centred care.

There are several potential unmeasured confounding variables to explain our findings. It is likely that the preference-sensitive nature of prostate cancer treatment decision-making is an important factor. Most notably among these is referral bias. Patients who receive radiation oncologist consultation may have expressed a preference for radiation treatment during the initial discussion of treatment options with their referring urologists. Conversely, those who are set on a surveillance strategy following consultation with a urologist may be less likely to undergo radiation oncologist consultation. In other words, patient preference of wanting radiation treatment would drive radiation oncology referrals, while patients who wish surveillance would decline a radiation oncology referral which could explain this association. Diagnosing urologist characteristics may also influence a patient’s likelihood of radiation oncology consultation^2^ or undergoing surveillance.^14^

Further, based on the referral itself from a urologist, patients may perceive the need for treatment by the radiation oncologist. As cancer organisations (such as the National Comprehensive Cancer Network) and government bodies (such as Cancer Care Ontario) mandate radiation oncology consultation for all newly diagnosed prostate cancer patients,^3, 4^ referral to radiation oncologists should be viewed by patients as a consultation and not a need for treatment. Given current guidelines,^15, 16^ such consultation should emphasize conservative management for patients with low-risk disease.

Jang et al.^17^ previously examined the effect of specialist and PCP visits on prostate cancer treatment choice and demonstrated that patients seen by urologists alone had the highest utilisation of conservative therapy, while those seen by urologists and radiation oncologists had the lowest. Notably, they found that PCP visits were associated with increased use of conservative management, while we found the opposite. More recently, Chamie et al. recently examined the use of surgery, radiotherapy and surveillance. Radiotherapy was the most commonly utilised treatment, with little correlation to tumour factors.^18^ However, patients were diagnosed between the mid-1990s and early 2000s (Jang) or during the mid-2000s (Chamie), during which time active surveillance was much less widely accepted. Second, they utilised SEER-Medicare linked data which have significant limitations when compared with the Ontario data. While SEER-Medicare data are limited to patients over the age of 65, we examined all patients regardless of age. Further, we captured all patients diagnosed in Ontario while SEER captures less than one-third of patients in the United States.^19^

Despite these strengths, there are limitations to our study. As we could only capture treatments reimbursed through the public health care system, it may underestimate active treatment. Patients who opt for and privately pay for non-standard treatments such as high intensity focused ultrasound and other modalities would be classified as receiving conservative management in this study. Also, there was no central pathology adjudication. However, in our subgroup analysis of patients undergoing biopsy at an academic facility with dedicated genitourinary pathologists, we observed consistent results indicating that upgrading on re-review is unlikely to explain the observed differences.

## Conclusions

In a large, contemporary, population-based cohort of patients newly diagnosed with prostate cancer, radiation oncology consultation prior to treatment decision was associated with an increased rate of active treatment. Selection and referral biases, and unmeasured confounding such as patient preference must be considered as important factors attributing this association. Multidisciplinary consultations should continue to be utilised after accounting for these biases and how patient preferences can impact decision-making.

### Availability of data and material

This study made use of de-identified data from the ICES Data Repository, which is managed by the Institute for Clinical Evaluative Sciences. The authors are unable to provide data without direct approval from the Institute for Clinical Evaluative Sciences.

## Electronic supplementary material

Supplementary Tables(DOCX 24 kb)
