# Supplementary material for: The effect of selection and referral biases for the treatment of localised prostate cancer with surgery or radiation
Source: Br J Cancer. 2018 Mar 29;118(10):1399–405. doi: 10.1038/s41416-018-0071-4 (PMC5959849; doi:10.1038/s41416-018-0071-4)
Supplement: Supplementary file 1 — Supplementary Tables(DOCX 24 kb) [file 41416_2018_71_MOESM1_ESM.docx]

**SUPPLEMENTARY APPENDIX**

| **Supplementary Table 1. Procedural codes used to define outcomes.** | | |
| --- | --- | --- |
| **Source** | **Code** | **Description** |
| **Radical prostatectomy** | | |
| OHIP | S651 | Retropubic prostatectomy, radical |
|  | S653 | Laparoscopic radical prostatectomy |
| CCI | 1.QT.91 | Excision radical, prostate |
| **Radiation therapy** | | |
| **Brachytherapy** | | |
| OHIP | S640 | Stereotactic prostate brachytherapy |
|  | X323 | Intracavitary application of radium or sealed sources, first application |
|  | X324 | Interstitial application of radium or sealed source radioisotope |
|  | X325 | Application of radium or radioisotope plaque or mould |
|  | J138 | Intracavitary ultrasound (TRUS) |
| CCI | 1.QT.26BAEB | Brachytherapy, prostate |
|  | 1.QT2.6BAEC | Brachytherapy, prostate |
|  | 1.QT.26HA | Brachytherapy, prostate |
|  | 1.QT.26HAEB | Brachytherapy, prostate |
|  | 1.QT.26HAEC | Brachytherapy, prostate |
|  | 1.QT.26LA | Brachytherapy, prostate |
|  | 1.QT.26LAEB | Brachytherapy, prostate |
|  | 1.QT.26LAEC | Brachytherapy, prostate |
|  | 1.QT.53HAEM | Implantation of internal device, prostate (brachytherapy applicator) |
|  | 1.QT.53LAEM | Implantation of internal device, prostate (brachytherapy applicator) |
| **External beam radiotherapy** | | |
| OHIP | X310 | Radiation treatment planning, Level 1 |
|  | X311 | Radiation treatment planning, Level 2 |
|  | X312 | Radiation treatment planning, Level 3 |
|  | X313 | Radiation treatment planning, Level 4 |
| CCI | 1.SQ.27JA | Radiation, prostate |
|  | 1.QT.27JA | Radiation, prostate |
|  | 1.QT2.7JADA | Radiation, prostate |
|  | 1.QT.27JADB | Radiation, prostate |
|  | 1.QT2.7JADC | Radiation, prostate |
|  | 1.QT.27JADE | Radiation, prostate |
|  | 1.QT.27JADG | Radiation, prostate |
|  | 1.QT.27JX | Radiation, prostate |

| **Supplementary Table 2. Treatment allocation for patients with low risk prostate cancer undergoing diagnostic prostate biopsy at an academic institution in the unmatched cohort.** | | |
| --- | --- | --- |
|  | RadOnc (n,%) | Urologist alone (n,%) |
| No treatment | 129 (49.2) | 506 (89.7) |
| Radiation | 121 (46.2) | 22 (3.9) |
| *EBRT* | *58 (22.1)* | *11 (2.0)* |
| *Brachytherapy** | *63 (24.1)* | *11 (2.0)* |
| Surgery | 12 (4.6) | 36 (6.4) |
| Abbreviations: EBRT = external beam radiotherapy.  * includes both low-dose rate (LDR) and high-dose rate (HDR) brachytherapy. | | |

| **Supplementary Table 3. Treatment allocation, stratified based on time of consultation with radiation oncologist, in the unmatched cohort.** | | | |
| --- | --- | --- | --- |
|  | **Same day RadOnc (n,%)** | **Asynchronous RadOnc (n,%)** | **No RadOnc (n,%)** |
| Whole cohort | | | |
| No treatment | 130 (20.3) | 998 (15.5) | 4591 (47.9) |
| Radiation | 276 (43.1) | 3882 (60.3) | 667 (6.9) |
| *EBRT* | *186 (29.1)* | *3228 (51.1)* | *523 (5.5)* |
| *Brachytherapy** | *90 (14.1)* | *654 (10.2)* | *144 (1.5)* |
| Surgery | 234 (36.6) | 1499 (23.3) | 4329 (45.2) |
| Low risk disease | | | |
| No treatment | 64 (56.1) | 384 (42.0) | 2293 (88.5) |
| Radiation | 39 (34.2) | 473 (51.7) | 113 (4.4) |
| *EBRT* | *14 (12.3)* | *198 (21.6)* | *43 (1.7)* |
| *Brachytherapy** | *25 (21.9)* | *275 (30.1)* | *70 (2.7)* |
| Surgery | 11 (9.7) | 58 (22.8) | 185 (7.1) |
| Intermediate/High risk disease | | | |
| No treatment | 66 (12.6) | 614 (11.1) | 2298 (32.9) |
| Radiation | 237 (45.1) | 3469 (62.8) | 554 (7.9) |
| *EBRT* | *172 (32.7)* | *3090 (55.9)* | *480 (6.9)* |
| *Brachytherapy** | *65 (12.4)* | *379 (6.9)* | *74 (1.1)* |
| Surgery | 223 (42.4) | 1441 (26.1) | 4144 (59.2) |
| Abbreviations: EBRT = external beam radiotherapy.  * includes both low-dose rate (LDR) and high-dose rate (HDR) brachytherapy. | | | |
